# Serum GM-CSF level is a predictor of treatment response to tocilizumab in rheumatoid arthritis patients: a prospective observational cohort study

**DOI:** 10.1186/s13075-024-03373-y

**Published:** 2024-07-12

**Authors:** Jingbo Su, Wenlu Hu, Yanxia Ding, Panpan Zhang, Tianfang Li, Shengyun Liu, Lihua Xing

**Affiliations:** 1 Department of Rheumatology and Immunology, The First Affiliated Hospital of Zhengzhou University, E. Jianshe Rd. 1, Zhengzhou, 450052 China; 2 Department of Respiratory, the First Affiliated Hospital of Zhengzhou University, E. Jianshe Rd. 1, Zhengzhou, 450052 China

**Keywords:** Rheumatoid arthritis, Tocilizumab, Predictors, Treatment response, GM-CSF

## Abstract

**Background:**

The aim of this prospective observational cohort study was to unveil the predictors of treatment response to tocilizumab (TCZ) therapy in rheumatoid arthritis (RA) patients, in terms of clinical characteristics and serum proinflammatory cytokines, especially to explore the predictive value of granulocyte macrophage-colony stimulating factor (GM-CSF).

**Methods:**

Active adult RA patients with inadequate response to MTX intending to receive TCZ therapy were recruited prospectively in the study. A total of 174 severe RA patients were included for the identification of the associations between treatment response and the following characteristic features: demographics, medications, disease activity, serum proinflammatory cytokines and so on.

**Results:**

Disease duration (OR = 0.996), tender joint count (TJC)/68 (OR = 0.943), neutrophil ratio (W4/baseline) (OR = 0.224), the high level of GM-CSF > 5 ng/ml (OR = 0.414) at baseline were the independent adverse predictors of good response assessed by clinical disease activity index (CDAI) at week 24 (W24) for TCZ therapy in RA patients. Moreover, DAS28-ESR (OR = 2.951, *P* = 0.002) and the high level of GM-CSF > 10 ng/ml at baseline (OR = 5.419, *P* = 0.002) were independent predictors of poor response, but not the high level of GM-CSF > 5 ng/ml (OR = 2.713, *P* = 0.054). The patients in the high GM-CSF group had significantly higher DAS28-ESR and serum levels of cytokines (IL-17A, IL-1β, IL-6, TNF-α) at baseline, as well as significantly higher rate of non-good response (62.8% vs. 39.4%, *P* = 0.010) and poor response (27.9% vs. 9.1%, *P* = 0.004) than the low GM-CSF group at W24. In addition, poor responders had significantly higher levels of GM-CSF with concomitant increase in the serum levels of IL-17A and IL-1β at baseline than those in moderate and good response groups, while serum levels of IL-6 and TNF-α at baseline were not significantly different in three response groups.

**Conclusion:**

The high levels of GM-CSF (> 5 ng/ml and > 10 ng/ml) at baseline were the independent predictors of non-good response and poor response to TCZ at W24 respectively. The high level of GM-CSF at baseline is a marker of high disease activity and a predictor of poor response to TCZ in severe RA patients, which may facilitate the development of individualized treatment strategies for refractory RA.

## Introduction

Rheumatoid arthritis (RA) patients may have different treatment responses to biologic disease-modifying anti-rheumatic drugs (bDMARDs), with roughly two-thirds of responders and one-third of non-responders [1]. Taking into consideration of the destructive nature of RA, the risk of adverse effects, the heterogeneous treatment responses, and the medical cost-effectiveness, it is imperative to identify predictors of treatment response before starting biotherapy. In fact, great endeavors have been made to unveil the predictors of the responses to interleukin (IL)-6 inhibitors. Previous studies have demonstrated that some clinical features may be related to treatment response of TCZ, including age [2], disease duration [3], number of previously-used DMARDs [4], tender joint count [2], acute phase reactants [2, 4], neutrophil ratio (W4/baseline) [5], and disease activity scores [2–5]. However, such associations are not always consistent, possibly due to the small sample size, retrospective nature of the study, selection bias of patients, different evaluation methods for disease activity, and the time points of assessments. As clinical characteristics are not completely reliable for individualized drug selection, we posited that the certain cytokines may have predictive values regarding the treatment response to bDMARDs.

Proinflammatory cytokines play a critical role in the pathogenesis and perpetuation of RA by inciting synovitis and systemic complications. The major cytokines include IL-6, tumor necrosis factor-α (TNF-α), granulocyte macrophage-colony stimulating factor (GM-CSF), IL-1, IL-23/IL-17, IL-8, IL-4/5 and type I interferon [6]. Some studies have been performed to identify the cytokine as predictors for the treatment response to TCZ in RA patients. However, they mainly focused on serum IL-6 and soluble IL-6 receptor (sIL-6R), and used 28-joint disease activity score (DAS28) to assess the disease activity, generating inconsistent results [7–12]. While patients with high levels of serum IL-17A [11] and IL-1β [13] at baseline had poor response to TCZ, but serum levels of TNF-α did not correlate treatment response to TCZ [11, 12]. Relatively small sample sizes make it difficult to get consistent and reproducible results, warranting larger prospective studies for the identification of reliable and convenient cytokine predictors for treatment response. Of note, GM-CSF is a crucial cytokine in the formation of chronic arthritis [14], and is abundant in synovial fluid and synovial tissue [15, 16]. However, no study has been carried out to evaluate the potential of serum GM-CSF to be a predictor for the treatment response to TCZ in RA patients so far.

Based on these observations, we conducted a prospective observational cohort study to analyze potential predictors of treatment response to TCZ in active adult RA patients. The serum levels of proinflammatory cytokines (IL-6, TNF-α, GM-CSF, IL-1β, IL-17A) were detected to validate the results of previous studies and explore the predictive value of GM-CSF.

## Methods

### Patients

Active adult RA patients with inadequate response to MTX treatment were recruited prospectively in the study from February 2021 to June 2022 in the First Affiliated Hospital of Zhengzhou University. Inclusion criteria were as follows: 1) All patients fulfilled the 2010 American College of Rheumatology/European League Against Rheumatism (ACR/EULAR) RA classification criteria, 2) DAS28-ESR ≥ 3.2 after MTX therapy at the maximum tolerated dose for more than three months, 3) optional concomitant stable doses of oral corticosteroids (prednisone ≤ 10 mg/d or equivalent) for more than four weeks, 4) patients were all TCZ-naïve and received no bDMARDs or Janus kinase inhibitor (JAKi) within three months. While patients with active infection, tumor, serious and unstable organ diseases were excluded.

Patients received intravenous TCZ 8mg/kg once every four weeks for 24 weeks, all combined with a stable dose of oral MTX. During the 24-week follow-up period, the doses of corticosteroids remained unchanged. In total, 180 RA patients were eligible at the baseline, six patients withdrew due to adverse events (three with liver dysfunction, one with thrombocytopenia, one with pneumonia, one with duodenal ulcer). Finally, 174 patients were included for the assessment of treatment response to TCZ.

### Evaluation of treatment response

TCZ therapy decreased ESR and CRP rapidly regardless of its clinical effectiveness, so DAS28-ESR, DAS28-CRP, and simplified disease activity index (SDAI) may overestimate its therapeutic effect and were not reliable measures for treatment response. Therefore, the clinical disease activity index (CDAI) was used to assess the disease activity of RA as it is a composite measure not using ESR and CRP [17].

Assessed by CDAI at W24, good response was defined as CDAI ≤ 10, including remission (≤ 2.8) and low disease activity (LDA, ≤ 10 and > 2.8). Moderate response was defined as moderate disease activity (MDA, ≤ 22 and > 10), and poor response was defined as high disease activity (HDA, > 22).

### Parameters associated with treatment response to TCZ

We investigated putative predictive factors (clinical or laboratory) of treatment response by CDAI at W24. Several parameters that could be related to different treatment responses were analyzed: age, gender, disease duration, previously-used DMARDs, combined glucocorticoid and MTX, neutrophils, neutrophil ratio (W4/baseline), rheumatoid factor (RF), anti-cyclic citrullinated peptide antibody (ACPA), ESR/CRP, tender or swollen joint count (TJC/SJC), health assessment questionnaire (HAQ) score, and disease activity scores (DAS28, CDAI, SDAI).

The crucial cytokines such as TNF-α, IL-6, GM-CSF, IL-17A, and IL-1β were tested in the serum of 142 patients at baseline, among them 95 patients were tested for serum cytokines at W12 and W24, using a multiplex bead immunoassay with Luminex laser based fluorescent analytical test instrumentation. Cytokine concentrations were calculated by reference to the standard curve. Values below the detection threshold [TNF-α (1.4%), IL-6 (24.6%), IL-1β (14.8%), GM-CSF (44.4%), IL-17A (35.9%)] were replaced by a value equal to half of the lowest limit of quantification to retain these values for the analysis. Besides detection bias caused by different methods and reagent kits, the cytokine concentrations in our study were largely consistent with prior researches [9, 11–13, 18]. Of note, our study mainly aimed to compare the cytokine concentration in different treatment response groups. The optimal cutoff points for the neutrophil ratio and proinflammatory cytokines were calculated using the receiver operating characteristic (ROC) curve. The cutoff value of neutrophil ratio (W4/baseline) was 0.75, and the cutoff values of cytokines were GM-CSF (5.0 ng/ml), IL-17A (5.0 ng/ml), and IL-1β (0.4 ng/ml).

### Statistical analysis

The sample size was estimated with the following assumptions: 1) a two-tailed alpha of 0.05, 2) the rate of good response assessed by CDAI at 24W was 34.0% ~ 53.5% according to the previous studies [13, 17], 3) a lost-to-follow-up rate of 20%. Performed by “Two-Sided Confidence Intervals for One Proportion” in PASS 2021 software, the calculated sample size was 102, and dropout-inflated enrollment sample size was 128. If a two-tailed alpha of 0.01, the calculated sample size was 170. Thus, we expected that our sample size of 174 would be sufficient for subsequent analyses.

Continuous data were described as mean ± standard deviation or median (interquartile range), while categorical variables were presented as number of cases with percentages. The T test or Mann–Whitney U test for continuous variables and Chi-square test or Fisher’s exact test for categorical variables were used for comparing two groups. Univariate logistic analysis was used to screen for potential predictive variables, and a multivariate regression model was generated for independent predictors to highlight the respective influence of each covariate on the endpoint. ROC curve was utilized to evaluate predictive ability, the area under the curve (AUC) provided a measure of the overall discriminative ability. Kruskal–Wallis H-test and Bonferroni t-test was applied to multiple comparisons in different treatment response groups. Correlations were assessed by the Spearman’s rank correlation analysis. *P* < 0.05 is statistical significance. Statistical analysis was performed using SPSS 26.0 software.

## Results

### The predictors of treatment response to TCZ

A total of 174 RA patients were included for the assessment of treatment response to TCZ, 173 patients had severe disease activity, only one patient had moderate disease activity (CDAI 20.8) at baseline. Assessed by CDAI at W24, the rates of different treatment responses were good response [93 (53.4%)], moderate response [53 (30.5%)], and poor response [28 (16.1%)]. Among 142 patients with cytokines at baseline, the rates of different treatment responses were good response [76 (53.5%)], moderate response [45 (31.7%)], and poor response [21 (14.8%)].

Compared with the good response group, the patients in the non-good response group significantly had older age, longer disease duration, more numbers of previously-used csDMARDs, neutrophil ratio (W4/baseline) > 0.75, and higher TJC/68, SJC/66, ESR, HAQ score and disease activity scores (Table 1). The rate of high GM-CSF level > 5 ng/ml was 30.4% at baseline. The rate of high GM-CSF level and serum GM-CSF level at baseline were significantly higher in the non-good response group (40.9% vs. 21.1%, *P* < 0.001) and [3.66 (1.0–11.99) pg/mL vs. 1.0 (1.0–4.71) pg/mL, *P* = 0.010], respectively. Serum levels of TNF-α, IL-6, IL-1β, IL-17A had no significant differences between the two groups, however the rates of high IL-17A level > 5 ng/ml and high IL-1β level > 0.4 ng/ml were increased in the non-good response group with slightly significant differences.

**Table 1:** Characteristics between good response group and non-good response group

Variable	Total (*n* = 174)	Good response (*n* = 93)	Non-good response (*n* = 81)	*P* value
**Demographics**				
Female, n (%)	149 (85.6)	80 (86.0)	69 (85.2)	0.875
Age at enrollment (years)	48.1 ± 11.0	46.2 ± 11.0	50.2 ± 10.7	0.016*
Disease duration (months)	67.0 (28.0–144.0)	51.0 (20.5–117.5)	102.0 (36.5–162.0)	0.001*
**Medications**				
Used bDMARDs/JAKi, n (%)	46 (26.4)	22 (23.7)	24 (29.6)	0.373
The number of previously-used csDMARDs	2 (1–3)	1 (1–2)	2 (1–4)	< 0.001*
Glucocorticoid use, n (%)	130 (74.7)	70 (75.3)	60 (74.1)	0.856
Prednisone equivalent doses (mg/day)	5.0 (0–5.0)	5.0 (1.25–5.0)	5.0 (0–5.0)	0.818
MTX doses (mg/week)	15.0 (15.0–15.0)	15.0 (15.0–15.0)	15.0 (15.0–15.0)	0.487
**Laboratory results**				
RF positive, n (%)	137/159 (86.2)	78/88 (88.6)	59/71 (83.1)	0.315
ACPA positive, n (%)	135/159 (84.9)	76/88 (86.4)	59/71 (83.1)	0.568
Baseline neutrophil counts, × 10^9^/L	4.85 (3.96–6.06)	4.86 (4.11–6.25)	4.76 (3.78–5.98)	0.442
Neutrophil ratio (W4/baseline)	0.66 (0.50–0.87)	0.62 (0.48–0.78)	0.74 (0.52–0.98)	0.010*
Neutrophil ratio (W4/baseline) ≤ 0.75, n (%)	107 (61.5)	66 (71.0)	41 (50.6)	0.006*
**Disease activity at baseline**				
ESR (mm/H)	35.0 (21.0–63.0)	31.0 (19.5–56.0)	40.0 (25.5–67.5)	0.040*
CRP (mg/L)	15.1 (5.9–44.4)	13.5 (5.5–42.8)	16.8 (5.9–46.0)	0.515
TJC/68	24 (15–39.25)	19 (13–31.5)	32 (20–43)	< 0.001*
SJC/66	15 (11–21)	13 (10–20)	18 (12–24)	0.002*
DAS28-ESR	6.25 ± 0.86	6.15 ± 0.84	6.76 ± 0.87	< 0.001*
DAS28-CRP	5.80 ± 0.82	5.68 ± 0.85	6.19 ± 0.82	< 0.001*
CDAI	39.01 ± 10.36	36.46 ± 10.50	44.00 ± 11.18	< 0.001*
SDAI	41.70 ± 11.06	39.40 ± 11.83	47.06 ± 12.25	0.001*
HAQ	1.03 ± 0.36	0.97 ± 0.36	1.13 ± 0.32	0.002*
**Serum cytokines at baseline**				
TNF-α (pg/mL)	4.52 (1.59–7.56)	4.04 (1.59–7.07)	4.77 (1.59–8.77)	0.457
IL-6 (pg/mL)	8.73 (0.13–22.95)	7.27 (0.22–22.76)	10.84 (0.13–23.67)	0.771
IL-1β (pg/mL)	0.32 (0.04–0.49)	0.31 (0.07–0.45)	0.33 (0.04–0.66)	0.175
high IL-1β level (> 0.4 ng/ml), n (%)	48/142 (33.8)	20/76 (26.3)	28/66 (42.4)	0.043*
IL-17A (pg/mL)	0.95 (0.20–4.31)	0.95 (0.20–3.77)	1.21 (0.20–4.45)	0.390
high IL-17A level (> 5 ng/ml), n (%)	21/142 (14.8)	7/76 (9.2)	14/66 (21.2)	0.044*
GM-CSF (pg/mL)	1.18 (1.0–6.50)	1.0 (1.0–4.71)	3.66 (1.0–11.99)	0.010*
high GM-CSF level (> 5 ng/ml), n (%)	43/142 (30.4)	16/76 (21.1)	27/66 (40.9)	< 0.001*
high GM-CSF level (> 10 ng/ml), n (%)	27/142 (19.0)	6/76 (7.9)	21/66 (31.8)	< 0.001*

*Abbreviations*: *bDMARDs* Biological disease-modifying antirheumatic drugs, *csDMARDs* Conventional synthetic disease- modifying antirheumatic drugs, *JAKi* Janus kinase inhibitor, *RF* Rheumatoid factor, *ACPA* Anti-cyclic citrullinated peptide antibody, *TJC* Tender joint count, *SJC* Swollen joint count, *HAQ* Health assessment questionnaire, *DAS28* 28-joint disease activity score, *CDAI* Clinical disease activity index, *SDAI* Simplified disease activity index, *TNF-α* Tumor necrosis factor-α, *GM-CSF* Granulocyte macrophage- colony stimulating factor, *IL-6* Interleukin-6, *IL-1β* Interleukin-1β, *IL-17A* Interleukin-17A

Results are given as mean ± standard deviation, median (interquartile range) or n (%)

Statistically significant results (* *P* < 0.05) are highlighted in bold

Multiple logistic regression analysis (Table 2) further elucidated that disease duration (OR = 0.996), TJC/68 (OR = 0.943), neutrophil ratio (W4/baseline) (OR = 0.224), the high level of GM-CSF > 5 ng/ml at baseline (OR = 0.414) were the independent adverse predictors of good response. The multivariable model was relatively stable, while only taking the place of the high level of GM-CSF > 5 ng/ml, the predictive value of the high level of GM-CSF > 10 ng/ml at baseline was (OR = 0.232), and the other parameters’ OR almost remained the same.

**Table 2:** Logistic regression analysis for good response to TCZ at W24

Variables	Univariate analysis		Multivariate analysis	
	**OR (95%CI)**	***P* value**	**OR (95%CI)**	***P* value**
**Clinical characteristics**				
Female	0.933 (0.361–2.413)	0.886		
Age at enrollment (years)	0.966 (0.939–0.994)	0.018*		
Disease duration (months)	0.996 (0.992–0.999)	0.021*	**0.996 (0.991–0.999)**	0.049*
the number of previously-used csDMARDs	0.633 (0.492–0.815)	< 0.001*		
used bDMARDs/JAKi	1.208 (0.567–2.576)	0.624		
Neutrophil ratio (W4/baseline)	0.289 (0.087–0.963)	0.043*	**0.224 (0.058–0.862)**	0.030*
**Disease activity at baseline**				
ESR	0.988 (0.977–0.999)	0.034*		
CRP	1.001 (0.991–1.012)	0.788		
TJC/68	0.948 (0.926–0.971)	< 0.001*	**0.943 (0.917–0.969)**	< 0.001*
SJC/66	0.938 (0.900–0.978)	0.003*		
DAS28-ESR	0.439 (0.300–0.641)	< 0.001*		
DAS28-CRP	0.489 (0.355–0.715)	< 0.001*		
CDAI	0.939 (0.911–0.967)	< 0.001*		
SDAI	0.949 (0.924–0.975)	< 0.001*		
HAQ	0.240 (0.095–0.603)	0.002*		
**Serum cytokines at baseline**				
IL-6	0.996 (0.982–1.011)	0.626		
TNF-α	0.946 (0.883–1.012)	0.107		
IL-1β	0.405 (0.172–0.950)	0.038*		
IL-17A	1.002 (0.944–1.063)	0.948		
(a) high GM-CSF level (> 5 ng/ml)	0.385 (0.184–0.806)	0.011*	**0.414 (0.182–0.944)**	0.036*
(b) high GM-CSF level (> 10 ng/ml)	0.184 (0.069–0.490)	0.001*	**0.232 (0.081–0.670)**	0.007*

Regarding the high level of GM-CSF, the multivariable model firstly included (a) the high level of GM-CSF (> 5 ng/ml), secondly included (b) the high level of GM-CSF (> 10 ng/ml)

Statistically significant results (* *P* < 0.05) are highlighted in bold

According to the multivariable model as in Table 2, multiple logistic regression analysis of poor response revealed that DAS28-ESR [OR = 2.951, 95%CI (1.486–5.857), *P* = 0.002] and the high level of GM-CSF > 10 ng/ml at baseline [OR = 5.419, 95%CI (1.867–15.728), *P* = 0.002] were independent predictors of poor response, but not the high level of GM-CSF > 5 ng/ml [OR = 2.713, 95%CI (0.984–7.478), *P* = 0.054].

**Fig. 1:**
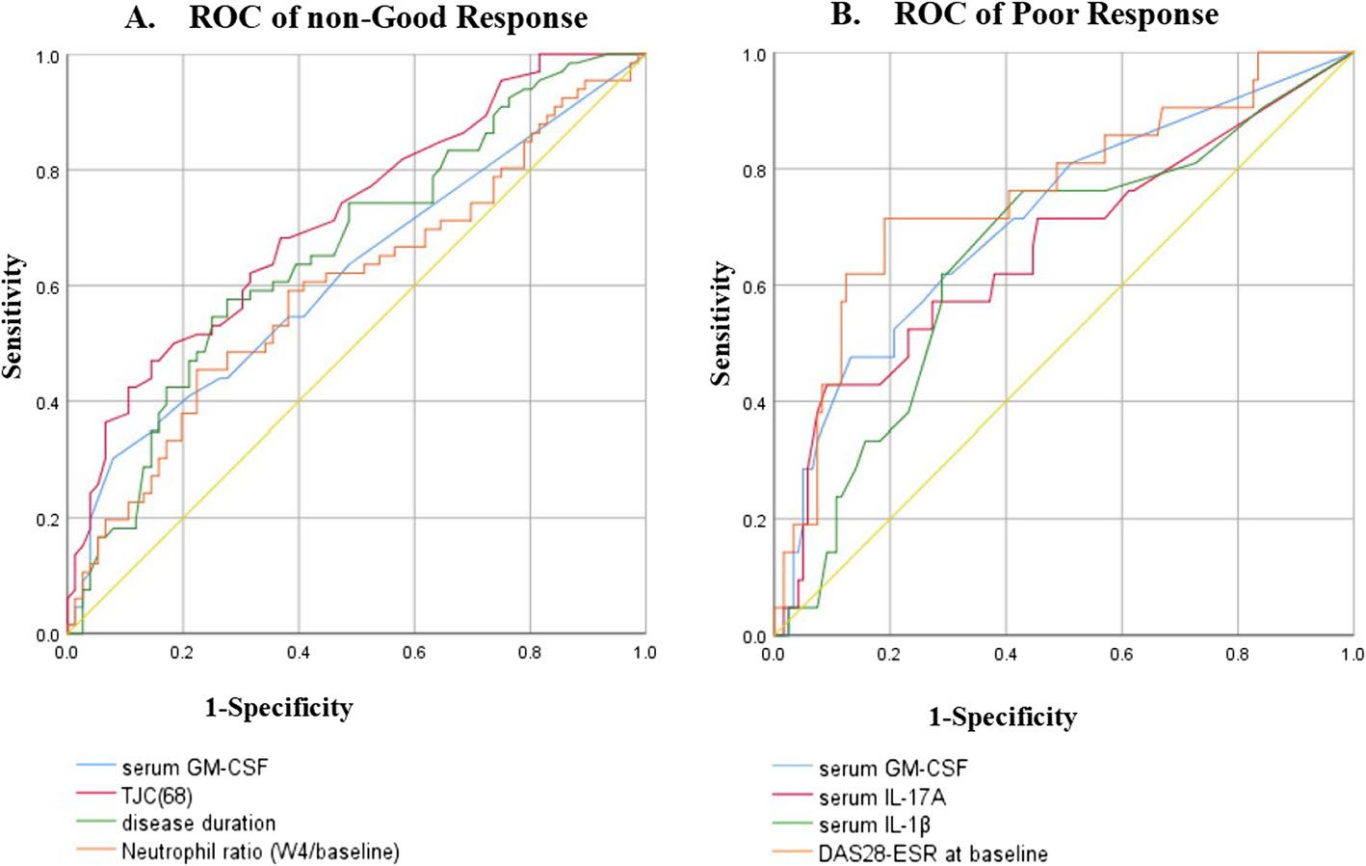
The ROC curve, the predictive ability for non-good response (**A**) and poor response (**B**) to TCZ at W24. The ROC curve analysis for non-good response (**A**), TJC/68 (AUC = 0.716), disease duration (AUC = 0.662), serum GM-CSF level (AUC = 0.619), neutrophil ratio (W4/baseline) (AUC = 0.598). The ROC curve analysis for poor response (**B**), DAS28-ESR (AUC = 0.758), serum GM-CSF level (AUC = 0.713), serum IL-17A level (AUC = 0.661), serum IL-1β level (AUC = 0.649)

The ROC curve analysis was conducted to evaluate the predictive value for non-good response (Fig. 1A), TJC/68 [AUC = 0.716, 95%CI (0.633–0.800), *P* < 0.001], disease duration [AUC = 0.662, 95%CI (0.572–0.751), *P* = 0.001], serum GM-CSF level [AUC = 0.619, 95%CI (0.526–0.713), *P* = 0.015], neutrophil ratio (W4/baseline) [AUC = 0.598, 95%CI (0.504–0.693), *P* = 0.044]. Meanwhile, the ROC curve analysis was conducted to evaluate the predictive value for poor response (Fig. 1B), DAS28-ESR [AUC = 0.758, 95%CI (0.637–0.878), *P* < 0.001], serum GM-CSF level [AUC = 0.713, 95%CI (0.589–0.838), *P* = 0.002], serum IL-17A level [AUC = 0.661, 95%CI (0.522–0.801), *P* = 0.018], serum IL-1β level [AUC = 0.649, 95%CI (0.519–0.779), *P* = 0.030].

### Comparison in different treatment responses to TCZ

As shown in Table 3, the patients in poor response group significantly had higher TJC/68, SJC/66, disease activity scores, serum levels of GM-CSF, and the rates of high GM-CSF level, high IL-17A level, high IL-1β level at baseline. Serum GM-CSF levels at baseline in three response groups were shown in Fig. 3F, poor responders had higher levels of GM-CSF significantly. However, significant differences were not detected in serum levels of IL-17A, IL-1β, TNF-α, IL-6, and neutrophil ratio (W4/baseline) in three response groups. In terms of clinical characteristics, disease duration, the number of previously-used csDMARDs, and neutrophil ratio (W4/baseline) can predict good response better, but not poor response.

**Table 3:** Characteristics between different treatment responses to TCZ at W24

Variables	Good response (Remission or LDA, *n* = 76)	Moderate response (MDA, *n* = 45)	Poor response (HDA, *n* = 21)	*P* value
**Clinical characteristics**				
Female, n (%)	65 (85.5)	39 (86.7)	18 (85.7)	0.985
Age at enrollment (years)	46.25 ± 10.98	49.18 ± 10.26	52.0 ± 12.18	0.075
Disease duration (months)	51.0 (20.5–117.5)	120.0 (36.5–168.0)	120.0 (54.0–168.0)	0.004*
Used bDMARDs/JAKi, n (%)	18 (23.7)	12 (26.7)	6 (28.6)	0.875
The number of previously-used csDMARDs	1.5 (1–2)	**2 (1–4) **^a^*	2 (1–4)	0.006*
Neutrophil ratio (W4/baseline)	0.63 (0.49–0.77)	0.75 (0.51–1.04)	0.69 (0.44–0.80)	0.078
Neutrophil ratio (W4/baseline) ≤ 0.75, n (%)	55 (72.4)	22 (48.9)	13 (61.9)	0.035*
**Disease activity at baseline**				
ESR (mm/H)	32.5 (18.8–51.3)	31.0 (21.0–48.0)	50.0 (28.5–73.0)	0.097
CRP (mg/L)	13.5 (6.0–44.7)	13.6 (7.1–38.1)	32.9 (4.2–70.0)	0.745
TJC/68	18.5 (12.3–31.5)	**27 (16.5–42.5) **^a^*	**39 (28.5–48.5) **^a^*	< 0.001*
SJC/66	13 (10.0–20.0)	15 (10.5–21.0)	**22 (14.5–26.5) **^a^*	0.001*
DAS28-ESR	6.13 ± 0.82	6.50 ± 0.86	**7.08 ± 0.84**^a^*	< 0.001*
DAS28-CRP	5.68 ± 0.83	6.01 ± 0.82	**6.46 ± 0.77 **^a^*	< 0.001*
CDAI	36.30 ± 10.20	**42.08 ± 11.69 **^a^*	**48.09 ± 10.85 **^a^*	< 0.001*
SDAI	39.21 ± 11.19	44.34 ± 12.06	**51.97 ± 11.64**^a^*	< 0.001*
HAQ	0.96 ± 0.36	1.13 ± 0.32	1.20 ± 0.29	0.004*
**Serum cytokines at baseline**				
TNF-α (pg/mL)	4.04 (1.59–7.07)	4.44 (1.59–7.93)	6.77 (1.69–10.44)	0.464
IL-6 (pg/mL)	7.27 (0.22–22.76)	8.73 (0.10–19.42)	14.43 (1.32–29.18)	0.453
IL-1β (pg/mL)	0.31 (0.07–0.45)	0.31 (0.04–0.57)	0.45 (0.25–0.77)	0.084
High IL-1β level (> 0.4 ng/ml), n (%)	20/76 (26.3)	15 (33.3)	**13 (61.9) **^a^*	0.010*
IL-17A (pg/mL)	0.95 (0.20–3.77)	0.95 (0.20–3.75)	4.02 (0.25–6.82)	0.053
High IL-17A level (> 5 ng/ml), n (%)	7/76 (9.2)	5 (11.1)	**9 (42.9) **^a^*^, b^*	< 0.001*
GM-CSF (pg/mL)	1.0 (1.0–4.71)	1.18 (1.0–8.72)	**6.38 (1.18–25.71)** ^a^*	0.002*
High GM-CSF level (> 5 ng/ml), n (%)	16 (21.1)	15 (33.3)	**12 (57.1) **^a^*	0.006*
High GM-CSF level (> 10 ng/ml), n (%)	6 (7.9)	10 (22.2)	**11 (52.4) **^a^*^, b^*	< 0.001*

Results are given as mean ± standard deviation, median (interquartile range) or n (%)

Statistically significant results (* *P* < 0.05, a*P < 0.017 vs. good response, b*P < 0.017 vs. moderate response) are highlighted in bold

### Comparison between high GM-CSF group and low GM-CSF group

**Fig. 2:**
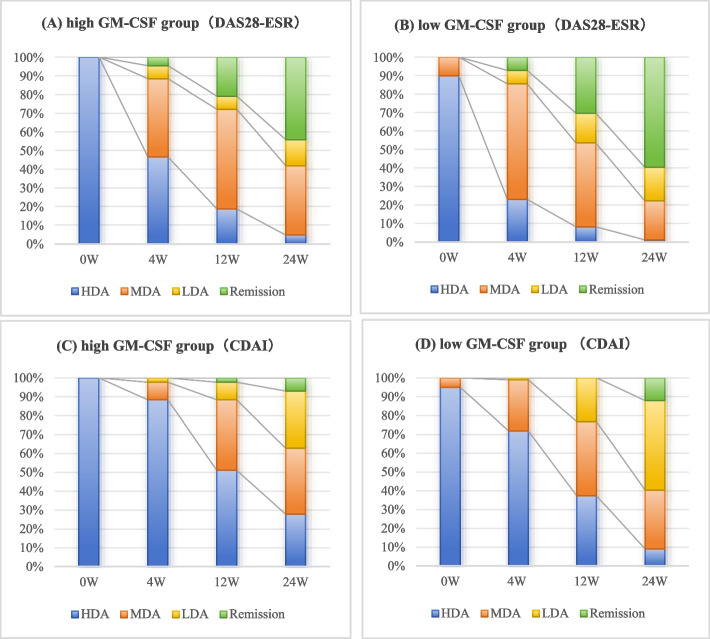
Treatment responses in different GM-CSF levels assessed by DAS28-ESR (**A**, **B**), and CDAI (**C**, **D**). Abbreviations: HDA high disease activity, MDA moderate disease activity, LDA low disease activity

The distribution of treatment responses in the high GM-CSF group and low GM-CSF group assessed by DAS28-ESR and CDAI respectively were shown in Fig. 2. Almost all patients had HDA at baseline, however the proportion of poor response (HDA) in the high GM-CSF group was higher than the low GM-CSF group at W4, W12 and W24. Compared with DAS28-ESR, CDAI is a more reliable measure for treatment response to TCZ. The patients in the high GM-CSF group had significantly higher rate of non-good response (MDA + HDA) and poor response (HDA) than the low GM-CSF group at W24, (62.8% vs. 39.4%, *P* = 0.010) and (27.9% vs. 9.1%, *P* = 0.004), respectively (Fig. 2 C, D).

Significant increases of ESR, DAS28-ESR, serum levels of cytokines (IL-17A, IL-1β, IL-6, and TNF-α) were demonstrated in patients with high GM-CSF levels compared with the low GM-CSF group at baseline (Table 4). Multiple logistic regression analysis further elucidated that DAS28-ESR (OR = 1.844), TNF-α (OR = 1.192), IL-1β (OR = 14.562) were the independent predictors of patients with high GM-CSF levels.

**Table 4:** Characteristics between high GM-CSF group and low GM-CSF group

Variables	High GM-CSF level (* n* = 43)	Low GM-CSF level (* n* = 99)	*P* value	Multivariate analysis for high GM-CSF	
				**OR (95%CI)**	***P* value**
**Demographics**					
Age at enrollment (years)	48.1 ± 12.8	48.0 ± 10.3	0.977		
Female, n (%)	37 (86.0)	85 (85.9)	0.976		
Disease duration (months)	70.0 (26.0–135.0)	78.0 (29.5–150.75)	0.749		
**Medications**					
used bDMARDs/JAKi, n (%)	12 (27.9)	24 (24.2)	0.645		
the number of previously-used csDMARDs	2 (1–4)	2 (1–3)	0.057		
Glucocorticoid use, n (%)	36 (83.7)	71 (71.7)	0.127		
Prednisone equivalent doses (mg/day)	5.0 (2.5–7.5)	5.0 (0–5.0)	0.050		
MTX doses (mg/week)	15.0 (15.0–17.5)	15.0 (15.0–15.0)	0.120		
**Laboratory results**					
RF positive, n (%)	27/31 (87.1)	80/96 (83.3)	0.829		
ACPA positive, n (%)	25/31 (80.6)	81/96 (84.4)	0.627		
Baseline Neutrophil counts, × 10^9^/L	4.63 (3.86–6.35)	4.88 (3.99–6.06)	0.912		
Neutrophil ratio (W4/baseline)	0.69 (0.52–0.89)	0.64 (0.49–0.80)	0.308		
**Disease activity at baseline**					
TJC/68	26 (16.25–43)	21 (15–37)	0.128		
SJC/66	16 (12–23.25)	15 (10–21)	0.230		
ESR (mm/H)	48.0 (30.0–65.3)	29.0 (18.0–46.0)	0.001*		
CRP (mg/L)	18.2 (5.1–45.4)	12.9 (6.1–39.3)	0.440		
DAS28-ESR	6.74 ± 0.82	6.24 ± 0.89	0.002*	**1.844 (1.079–3.153)**	0.025*
DAS28-CRP	6.09 ± 0.78	5.82 ± 0.89	0.078		
CDAI	41.88 ± 11.74	39.0 ± 11.40	0.174		
SDAI	44.87 ± 12.39	41.79 ± 12.22	0.171		
HAQ	1.08 ± 0.35	1.04 ± 0.35	0.615		
**Serum cytokines at baseline**					
TNF-α (pg/mL)	7.50 (4.59–12.14)	2.89 (1.59–6.33)	< 0.001*	**1.192 (1.067–1.333)**	0.002*
IL-6 (pg/mL)	14.55 (6.78–25.41)	4.61 (0.10–21.07)	0.004*		
IL-1β (pg/mL)	0.49 (0.32–0.96)	0.17 (0.04–0.33)	< 0.001*	**14.562 (3.290–64.446)**	< 0.001*
high IL-1β level (> 0.4 ng/ml), n (%)	28 (65.1)	20 (20.2)	< 0.001*		
IL-17A (pg/mL)	4.31 (1.17–5.93)	0.94 (0.20–2.46)	< 0.001*		
high IL-17A level (> 5 ng/ml), n (%)	16 (37.2)	5 (5.1)	< 0.001*		

Results are given as mean ± standard deviation, median (interquartile range) or n (%)

Statistically significant results (* *P* < 0.05) are highlighted in bold

### Correlation analysis of disease activity and cytokines at baseline

Spearman’s correlation analysis (Table 5) revealed that serum GM-CSF significantly correlated with ESR, DAS28-ESR, IL-1β, IL-6, and IL-17A at baseline. In addition, serum IL-1β correlated with ESR and IL-6, serum IL-17A correlated with TNF-α, and serum IL-6 correlated with ESR and CRP.

**Table 5:** Spearman’s correlation coefficients of disease activity and cytokines at baseline

Variables	ESR	CRP	TJC/68	SJC/66	DAS28-ESR	DAS28-CRP	CDAI	SDAI	HAQ	GM-CSF	IL-1β	IL-17A	IL-6
GM-CSF	0.360**	0.181*	0.183*	0.210*	0.308**	0.217**	0.144	0.163	0.010	-			
IL-1β	0.310**	0.181*	0.151	0.252**	0.283**	0.211*	0.178*	0.184*	0.012	0.634**	-		
IL-17A	0.152	0.086	0.071	0.108	0.125	0.105	0.107	0.119	-0.047	0.313**	0.294**	-	
IL-6	0.466**	0.423**	0.034	0.103	0.264**	0.235**	0.042	0.144	0.086	0.375**	0.439**	0.263**	-
TNF-α	0.047	0.011	-0.042	-0.007	0.016	-0.004	0.016	0.018	-0.154	0.255**	0.256**	0.747**	0.233**

Statistically significant results (* *P* < 0.05, ** *P* < 0.01 and Spearman’s correlation coefficients > 0.3) are highlighted in bold

### Cytokine alterations post TCZ treatment

**Fig. 3:**
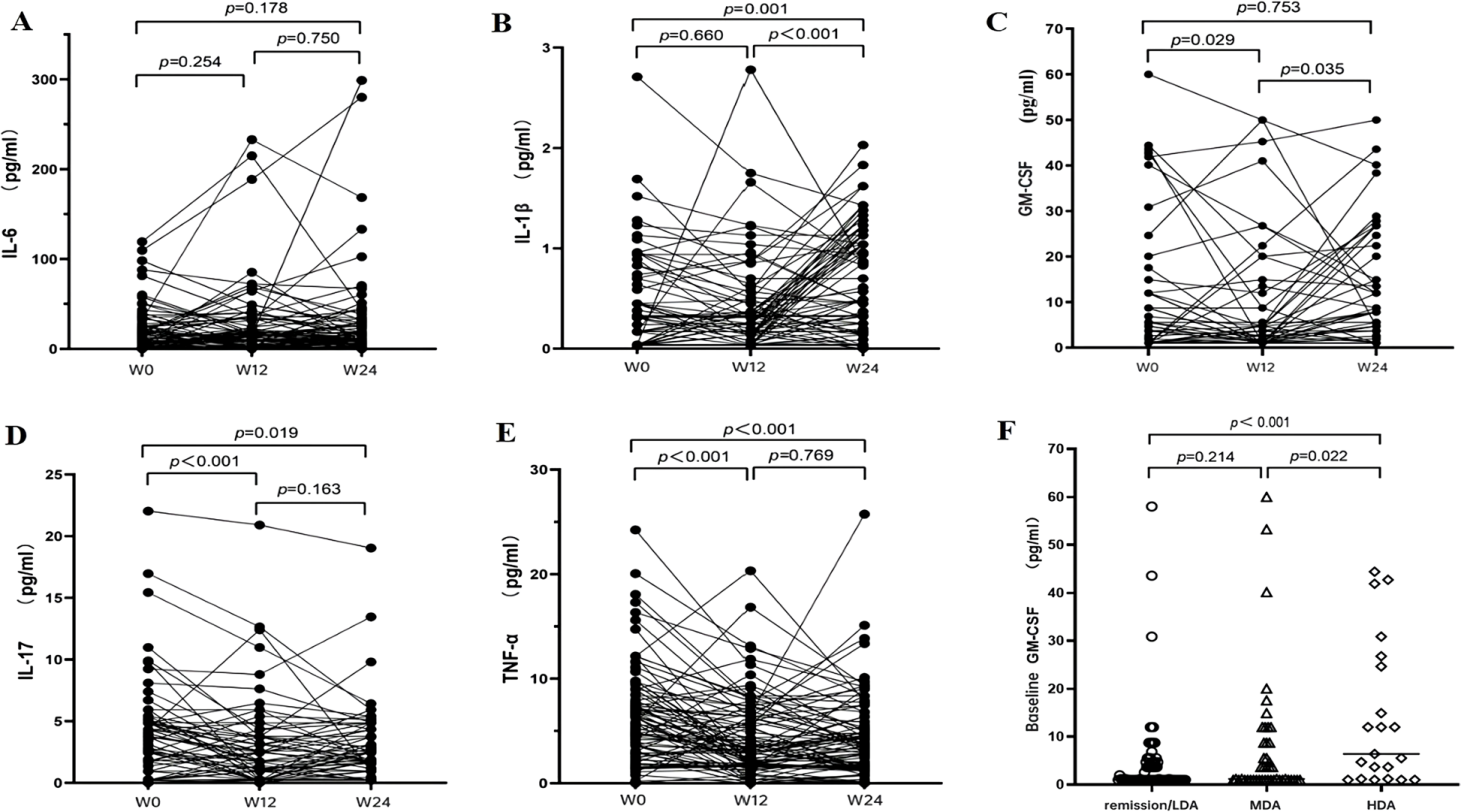
Cytokine alterations post TCZ treatment, including IL-6 (**A**), IL-1β (**B**), GM-CSF (**C**), IL-17A (**D**), TNF-α (**E**). Assessed by CDAI at W24, poor responders had significantly higher levels of GM-CSF at baseline (**F**)

Cytokine alterations among 95 patients at W0, W12 and W24 were shown in Fig. 3. Somewhat surprisingly, no significant changes were detected in the levels of IL-6 post TCZ treatment (Fig. 3A). Significant changes in the levels of IL-1β were not detected at W12, but significant increase was clearly seen at W24 (Fig. 3B). Serum GM-CSF levels decreased significantly at W12, but increased significantly at W24 (Fig. 3C). The IL-17A level significantly decreased at W12, while increased at W24 with no significant differences (Fig. 3D). The TNF-α levels significantly decreased at W12, but no significant differences between W12 and W24 (Fig. 3E).

## Discussion

This prospective observational cohort study was designed to identify predictors of treatment response to TCZ therapy in severe RA patients who had an inadequate response to MTX. Consistent with previous studies [2–5], disease duration, neutrophil ratio (W4/baseline), TJC, and DAS28-ESR are adverse predictors of good response assessed by CDAI at W24 for TCZ therapy in RA. To the best of our knowledge, this is the first report that demonstrates the high levels of GM-CSF (> 5 ng/ml and > 10 ng/ml) at baseline are the independent predictors of non-good response (MDA + HDA) and poor response (HDA) to TCZ at W24 respectively. The patients in the high GM-CSF group had significantly higher DAS28-ESR and serum levels of cytokines (IL-17A, IL-1β, IL-6, TNF-α) at baseline. In addition, poor responders had significantly higher levels of GM-CSF with concomitant increase in the serum levels of IL-17A and IL-1β at baseline.

In addition to its well-known hematopoietic role, GM-CSF plays an important role in the modulation of differentiation, polarization, and activation of immune cells such as neutrophils, macrophages, dendritic cells, and lymphocytes, which enhances typical immune/inflammatory cascade of chronic autoimmune diseases [19]. The various myeloid cellular responses (survival, proliferation, activation, and/or differentiation) that occur at different GM-CSF concentrations appear to be explained by a dose-dependent sequential model of GM-CSF receptor (GM-CSFR) activation [20]. GM-CSF extends neutrophil survival, primes the neutrophil oxidative burst, enhances phagocytosis and the formation of neutrophil extracellular traps (NETs) [21]. Moreover, GM-CSF may induce the polarization of synovial macrophages and activated M1 macrophages produce cytokines such as GM-CSF, TNF-α, IL-6, IL-1β, and IL-23 [21, 22]. GM-CSF is abundant in synovial fluid and synovial tissue [15, 16], which is a reasonable observation to explain highly activated macrophages in RA joints, suggesting a role in macrophage activation in joints that eventually leads to RA pathogenesis. Therefore, GM-CSF is a crucial cytokine in the formation of chronic arthritis [14], however, no previous study has been carried out to evaluate the potential of serum GM-CSF to be a predictor for treatment response to TCZ. Interestingly, 14–3-3η may be a valuable marker for the diagnosis of RA patients and it may have prognostic value [23]. 14–3-3η is a proinflammatory mediator critical to joint destruction in RA, serum 14–3-3η level is associated with high disease activity, joint erosion and destruction, and failure of remission [24]. 14–3-3 scaffold protein is a downstream binding protein for GM-CSF signaling pathway [25], and 14–3-3η belongs to them involved in a wide range of cellular functions. Over all, the above studies actually from some sides supports our findings that high GM-CSF level is associated with high disease activity, and a predictor of poor treatment response to TCZ. Further studies are required to confirm the correlation between serum GM-CSF and 14–3-3η, and explore the predictive value of them for treatment response to TCZ.

Because myeloid cell populations seem to be the main targets of GM-CSF activity during inflammation, the functions of GM-CSF are likely to be more restricted than those of proinflammatory cytokines with relatively broad effects, such as IL-6, TNF-α [26]. These functional differences indicate that GM-CSF may be a unique therapeutic target. While preclinical studies have demonstrated that GM-CSF inhibitors may repress inflammatory arthritis and alleviate pain, clinical efficacy trials of monoclonal antibodies targeting GM-CSF or GM-CSFR in RA patients have generated mixed results, inferiority to tofacitinib, sarilumab, and golimumab [27–29]. Notably, the effects of high doses of systemically administered GM-CSF on a disease may not necessarily be informative about the role of endogenous, potentially locally acting, GM-CSF in that disease. We showed that the rate of high GM-CSF level was only 30.4% at baseline among severe RA patients, possibly due to the different tissue distribution of GM-CSF. As mentioned above, GM-CSF mainly exists in synovial fluid and synovial tissue [15, 16], resulting in reduced serum levels. The available GM-CSF levels in an inflamed tissue at a particular time point may determine the nature of these pathways and whether GM-CSF can overflow into the circulation [20]. Such levels may also impact in turn on the effectiveness and route of administration of GM-CSF inhibitors. GM-CSF inhibitors maybe achieve better results administered by intra-articular injection, or administered in severe and refractory RA patients.

We first demonstrate that the serum levels of GM-CSF and IL-17A changed consistently post TCZ therapy in RA patients, which significantly decreased at W12, while increased at W24. Proinflammatory cytokines (IL-6, IL-1 and IL-23) are well characterized as an enhancer and stabilizer of effector Th17 cells [30, 31]. Th17 cells orchestrate a “GM-CSF-cytokine network” in forming chronic joint inflammation in SKG mice [14]. Arthritogenic Th17 cells stimulate fibroblast-like synoviocytes (FLSs) via IL-17 and promote GM-CSF production, which subsequently stimulate GM-CSF-producing synovial innate lymphoid cells (ILCs) in joints [14, 21]. Thus, the pathogenesis of Th17 cells in RA may shift from “IL-17-producer”, as an initiator of the disease, into “GM-CSF-producer”, as an organizer of chronic inflammation [32, 33]. Collectively, we posit that blocking IL-6R signaling pathway by TCZ, serum levels of TNF-a, IL-17A and GM-CSF are decreased in a short time after TCZ treatment, then the function of Th17 cells is inhibited. However, it will subsequently upregulate the level of IL-1β, a proinflammatory factor upstream of Th17 cells, increasing production of effector cytokines IL-17 and GM-CSF. In addition, poor responders had significantly higher levels of GM-CSF with concomitant increase in the serum levels of IL-17A and IL-1β at baseline, furtherly illuminate the pathogenicity of Th17 cells in RA. Accordantly with previous studies [11, 13], we also show correlations between the high levels of IL-17A and IL-1β with poor response to TCZ. Of note, correlations between cytokines also corroborates the above theories, there were significantly correlations among IL-1β, IL-6 and GM-CSF mutually, and also between IL-17A and GM-CSF, TNF-α.

Somewhat surprisingly, serum levels of IL-6 and TNF-α at baseline are not related to treatment response, with no significant differences in three response groups. Our findings are consistent with the previous studies, serum levels of TNF-α [11, 12] and IL-6 [10, 11] did not correlate treatment response to TCZ, in contradiction to some reports that high IL-6 level was related to good response [7–9] and poor response [12]. A plausible explanation for such discrepancy is that previous studies used unreliable DAS28 to assess the disease activity, as well as selection bias of patients. Almost all patients in our study have high disease activity at baseline, no significant differences were detected in serum level of IL-6 in three response groups and post TCZ treatment, suggesting that IL-6 is abundant in both “resolving” and “persistent” synovitis [6], and that dysregulated (excessive or persistent) IL-6 production causes acute and chronic immune disorders.

Neutrophil ratio (W4/baseline) ≤ 0.75 was an independent predictor of good response at W24 for TCZ therapy, but the predictive value was lower (AUC < 0.7). Moreover, neutrophil ratio (W4/baseline) had no significant differences in three response groups. In addition, the correlations between neutrophil ratio (W4/baseline) and treatment response were inconsistent in previous studies [2, 5], which may be attributed to selection bias of patients, different evaluation indicators to disease activity, and the concomitant use of glucocorticoid. Based on these observations, neutrophil ratio (W4/baseline) was not a reliable predictor for treatment response to TCZ. Neutrophils affect the various stages of RA pathogenesis and progression, from breaking immune tolerance to driving synovial joint inflammation [34]. Activated neutrophils are a major source of soluble IL-6 receptor, IL-6 stimulates the adherence of neutrophils to fibroblasts, the maturation and activation of osteoclasts, and synovial proliferation, all of which are crucial for the synovial pannus formation and bone destruction. A transition from neutrophil to monocyte accumulation at the site of inflammation suggests not only monocyte recruitment but also neutrophils disappearance [35–37]. However, the mechanism of neutropenia regarding TCZ therapy remains elusive, further studies are needed to determine the role of TCZ on neutrophil migration in RA patients.

As this was a prospective observational single-centered cohort study, it has several limitations, such as selection bias of patients and limited sample size. For instance, almost all patients were severe RA, so our findings may not be applied to RA patients with low and moderate activities. Second, the absence of other biotherapy control group makes it unclear whether the observed results are specific to tocilizumab or if similar outcomes could be achieved with other biological therapies. Finally, GM-CSF has limited application to clinical practice because of detection bias, it is imperative to establish a more reliable and convenient detection method for GM-CSF. Further validation through larger-sized prospective studies is required to confirm our results. Nevertheless, our observations may lend some novel insights to the pathogenesis of RA and to predict the treatment response to TCZ, which may facilitate the development of individualized treatment strategies for refractory RA.

## Conclusions

The high levels of GM-CSF (> 5 ng/ml and > 10 ng/ml) at baseline were the independent predictors of non-good response and poor response to TCZ at W24 respectively. The high level of GM-CSF at baseline is a marker of high disease activity and a predictor of poor response to TCZ in severe RA patients, which may facilitate the development of individualized treatment strategies for refractory RA.

## Data Availability

No datasets were generated or analysed during the current study.

## Abbreviations

bDMARDs: Biological disease-modifying antirheumatic drugs
csDMARDs: Conventional synthetic disease-modifying antirheumatic drugs
JAKi: Janus kinase inhibitor
RF: Rheumatoid factor
ACPA: Anti-cyclic citrullinated peptide antibody
TJC: Tender joint count
SJC: Swollen joint count
ESR: Erythrocyte sedimentation rate
CRP: C-reactive protein
HAQ: Health assessment questionnaire
DAS28: 28-Joint disease activity score
CDAI: Clinical disease activity index
SDAI: Simplified disease activity index
LDA: Low disease activity
MDA: Moderate disease activity
HDA: High disease activity
TNF-α: Tumor necrosis factor-α
GM-CSF: Granulocyte macrophage-colony stimulating factor
IL-6: Interleukin-6
sIL-6R: Soluble IL-6 receptor
IL-1β: Interleukin-1β
IL-17A: Interleukin-17A
NETs: Neutrophil extracellular traps
FLSs: Fibroblast-like synoviocytes
ILCs: Innate lymphoid cells
